# Applying the Principles for Digital Development: Case Study of a Smartphone App to Support Collaborative Care for Rural Patients With Posttraumatic Stress Disorder or Bipolar Disorder

**DOI:** 10.2196/10048

**Published:** 2018-06-06

**Authors:** Amy M Bauer, Sarah Hodsdon, Jared M Bechtel, John C Fortney

**Affiliations:** ^1^ Department of Psychiatry and Behavioral Sciences University of Washington Seattle, WA United States; ^2^ John Snow, Inc Boston, MA United States; ^3^ Health Services Research and Development Service Center of Innovation for Veteran-Centered and Value-Driven Care VA Puget Sound Health Care System Seattle, WA United States

**Keywords:** mHealth, mental health, primary health care, rural health, post-traumatic stress disorders, PTSD, bipolar disorder, depression

## Abstract

**Background:**

Despite a proliferation of patient-facing mobile apps for mental disorders, there is little literature guiding efforts to incorporate mobile tools into clinical care delivery and integrate patient-generated data into care processes for patients with complex psychiatric disorders.

**Objective:**

The aim of this study was to seek to gain an understanding of how to incorporate a patient-provider mobile health (mHealth) platform to support the delivery of integrated primary care–based mental health services (Collaborative Care) to rural patients with posttraumatic stress disorder and/or bipolar disorder.

**Methods:**

Using the Principles for Digital Development as a framework, we describe our experience designing, developing, and deploying a mobile system to support Collaborative Care. The system consists of a patient-facing smartphone app that integrates with a Web-based clinical patient registry used by behavioral health care managers and consulting psychiatrists. Throughout development, we engaged representatives from the system’s two user types: (1) providers, who use the Web-based registry and (2) patients, who directly use the mobile app. We extracted mobile metadata to describe the early adoption and use of the system by care managers and patients and report preliminary results from an in-app patient feedback survey that includes a System Usability Scale (SUS).

**Results:**

Each of the nine Principles for Digital Development is illustrated with examples. The first 10 patients to use the smartphone app have completed symptom measures on average every 14 days over an average period of 20 weeks. The mean SUS score at week 8 among four patients who completed this measure was 91.9 (range 72.5-100). We present lessons learned about the technical and training requirements for integration into practice that can inform future efforts to incorporate health technologies to improve care for patients with psychiatric conditions.

**Conclusions:**

Adhering to the Principles for Digital Development, we created and deployed an mHealth system to support Collaborative Care for patients with complex psychiatric conditions in rural health centers. Preliminary data among the initial users support high system usability and show promise for sustained use. On the basis of our experience, we propose five additional principles to extend this framework and inform future efforts to incorporate health technologies to improve care for patients with psychiatric conditions: design for public health impact, add value for all users, test the product and the process, acknowledge disruption, and anticipate variability.

## Introduction

### Background

Although there are thousands of smartphone apps for mental health conditions available on the marketplace [1], mobile tools have yet to have a substantial impact in the delivery of mental health services [2]. The majority of these tools are stand-alone, patient-facing, self-help apps that have not been subject to any evaluation or regulation. Research has demonstrated that very few people sustain use of such self-guided interventions [3-7], which has prompted clinical investigators to explore new models of technology-supported care [5,8-10]. Efforts to incorporate mobile tools into clinical care delivery and integrate patient-generated data into care processes are emerging. However, there is little research to guide these efforts, and few existing tools have demonstrated the capacity to link patients and providers directly [10-12]. Early experience suggests incorporating new health information technology tools into clinical practice is potentially disruptive because it directly impacts providers’ task behaviors and requires them to alter routines [13,14]. Adoption of health technologies in general, and patient-facing technologies in particular, thus has lagged [15].

### Objectives

To gain an understanding of how to incorporate a patient-provider mobile health (mHealth) platform into the clinical care of rural patients with posttraumatic stress disorder (PTSD) and bipolar disorder, we designed, developed, and deployed a smartphone app that is currently in use within the context of a large clinical trial, the Study to Promote Innovation in Rural Integrated Telepsychiatry (SPIRIT; NCT02738944). SPIRIT is a pragmatic trial by design, and therefore, the procedures and workflow are designed to approximate real-world practice [16-18]. The SPIRIT app supports the primary care–based delivery of mental health services through an evidence-based model of treatment delivery called Collaborative Care, described in additional detail below, which is the intervention offered in one arm of the treatment trial.

Here, we describe how the Principles for Digital Development [19,20], a set of best practices informing the development of technology-enabled programs, apply to the design and deployment processes for the SPIRIT app. We report early data on adoption of the SPIRIT app by care managers and patients, patients’ feedback on their use of the SPIRIT app, and lessons learned from the deployment process. This report focuses on the technology and its integration into the clinical model and is not an evaluation of effectiveness of the overall technology-supported care model. Recognizing that the requirements for mHealth tools differ from general requirements for mobile development [11], we propose extensions to the Principles for Digital Development for mHealth tools that are intended to support clinical services. We also propose the development of guidelines for mental health–specific digital design and user experience best practices.

## Methods

### Clinical Context

The SPIRIT app mobile system was designed to support the effective delivery of Collaborative Care. Collaborative Care is a model for delivering treatments for common mental disorders in primary care settings that is twice as effective as usual depression care [21-23]. For nearly two decades, it has been regarded as a best practice and has been widely disseminated [24-26]. Collaborative Care is defined by core principles that specify that care is team-based and patient-centered. Additionally, Collaborative Care provides measurement-based treatment-to-target, an evidence-based practice that involves routinely monitoring patient outcomes with standardized measures (eg, the Patient Health Questionnaire-9, PHQ-9 [27] for depression) and adjusting treatments when a patient is not improving [28]. Collaborative Care teams provide population-based care, which means providing care manager outreach to all of the patients in a defined population, not just those who show up for clinic visits [29]. This is in contrast to usual primary care in which patients often *fall through the cracks.* The detection and treatment of PTSD and bipolar disorder in primary care is poor [30-38]. Moreover, care is not measurement-based, follow-up is infrequent and ad hoc, and there is no proactive outreach [28,39].

**Figure 1:**
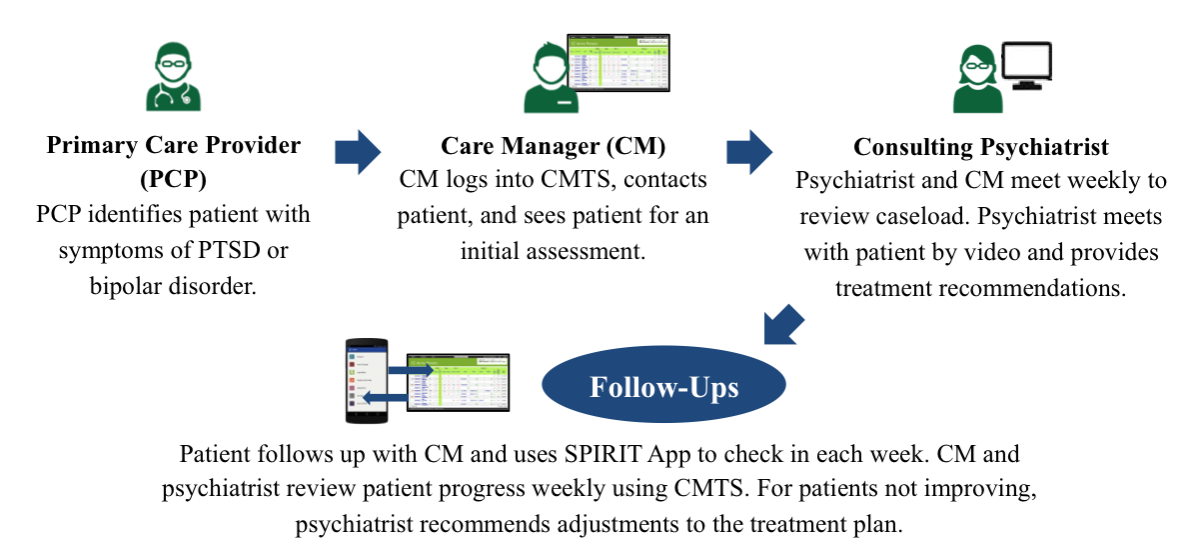
Study to Promote Innovation in Rural Integrated Telepsychiatry (SPIRIT) Collaborative Care workflow. CMTS: Care Management Tracking System; PTSD: posttraumatic stress disorder.

To support the Collaborative Care workflow (Figure 1), the University of Washington developed a Web-based patient registry that providers use to track patient visits and outcomes using standardized measures. The registry differs from an electronic health record (EHR) in its design for effective management of an entire patient population, key features that EHRs lack, and specific support for providers’ workflows. The registry, named the Care Management Tracking System (CMTS), supports workflows through reminders for proactive outreach and follow-up for patients who are not engaging in care and flags for patients who have not improved and may benefit from treatment changes.

The SPIRIT app was designed to provide a patient interface with CMTS, which is an entirely clinician-facing tool, and support patient’s own self-management and communication with the care manager. Given that empowering patients to improve self-management is a key goal of Collaborative Care, engaging patients in the use of digital technology to facilitate effective, whole-person care is a good fit for the Collaborative Care model [12,40,41]. Potential benefits of the SPIRIT app include improving patient engagement, increasing satisfaction by offering a convenient, asynchronous method for patient-provider communication, and enhancing measurement-based care through timely remote symptom monitoring. Providers may experience improved efficiency through reduced documentation because patients enter their own symptom scores and through reduced need for time-consuming synchronous telephone outreach and follow-up. In addition, the SPIRIT app was designed to increase the capacity of care managers so they can enroll more patients on their panel. By automating some of their clinical activities and helping them prioritize patients for outreach, the SPIRIT app is intended to increase the reach of the care manager and have a greater impact on population health.

### Mobile System Description

**Figure 2:**
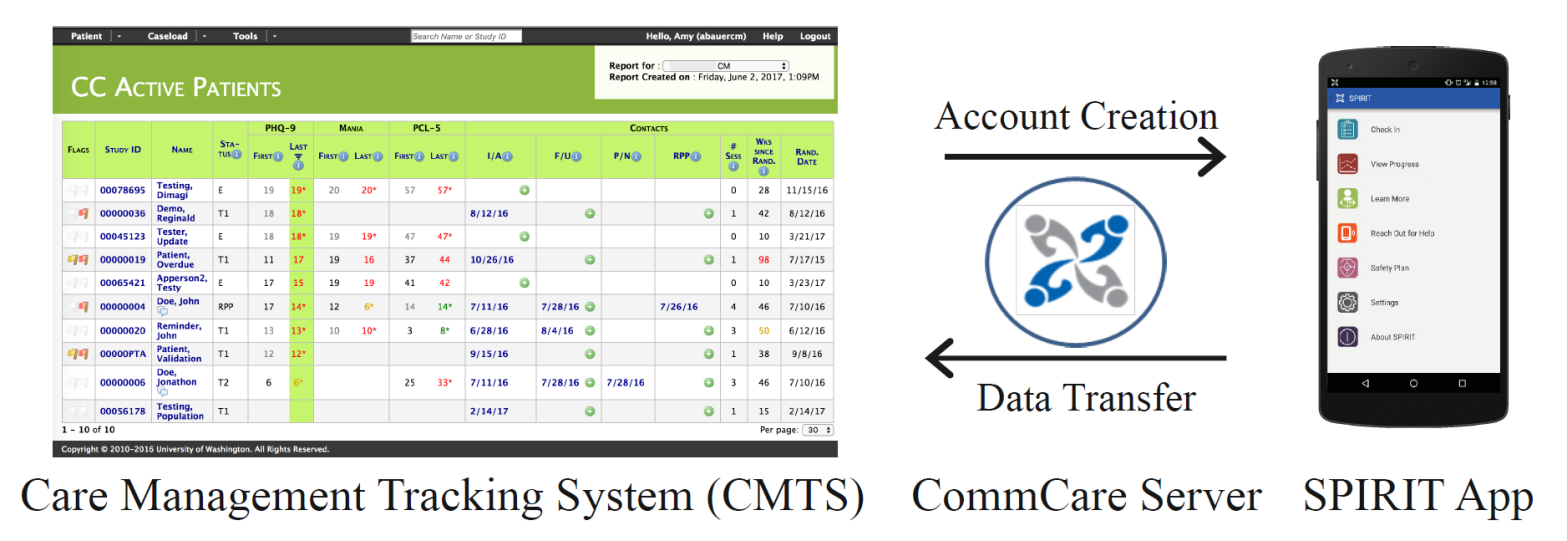
Diagram of the Study to Promote Innovation in Rural Integrated Telepsychiatry (SPIRIT) app mobile system.

The SPIRIT app is a free, password-protected Android app that securely transmits patient-generated data from a patient’s smartphone to the care manager through CMTS (Figure 2). It supports two user types: (1) patients, the primary users of the mobile app and (2) providers, who receive outputs from the mobile app on an online patient management system. The SPIRIT app was developed on CommCare, an open source, Software-as-a-Service (SaaS) mobile data collection platform. CommCare was selected for SPIRIT app development based on its strong evidence base in mHealth [42], robust case management functionality, and its turnkey application builder—which provided a graphical user interface (UI) to enable rapid agile design of the system. Data from the SPIRIT app is transmitted securely with encryption standards consistent with the Health Insurance Portability and Accountability Act (HIPAA) of 1996 from the mobile device to the CommCare server. CMTS pulls newly uploaded patient data from the CommCare server via an application programming interface hourly, or immediately if requested by the provider. The SPIRIT app is available in English and Spanish. Details of the system have been previously published [43] and are summarized below.

#### Patient Interface: Study to Promote Innovation in Rural Integrated Telepsychiatry App

**Figure 3:**
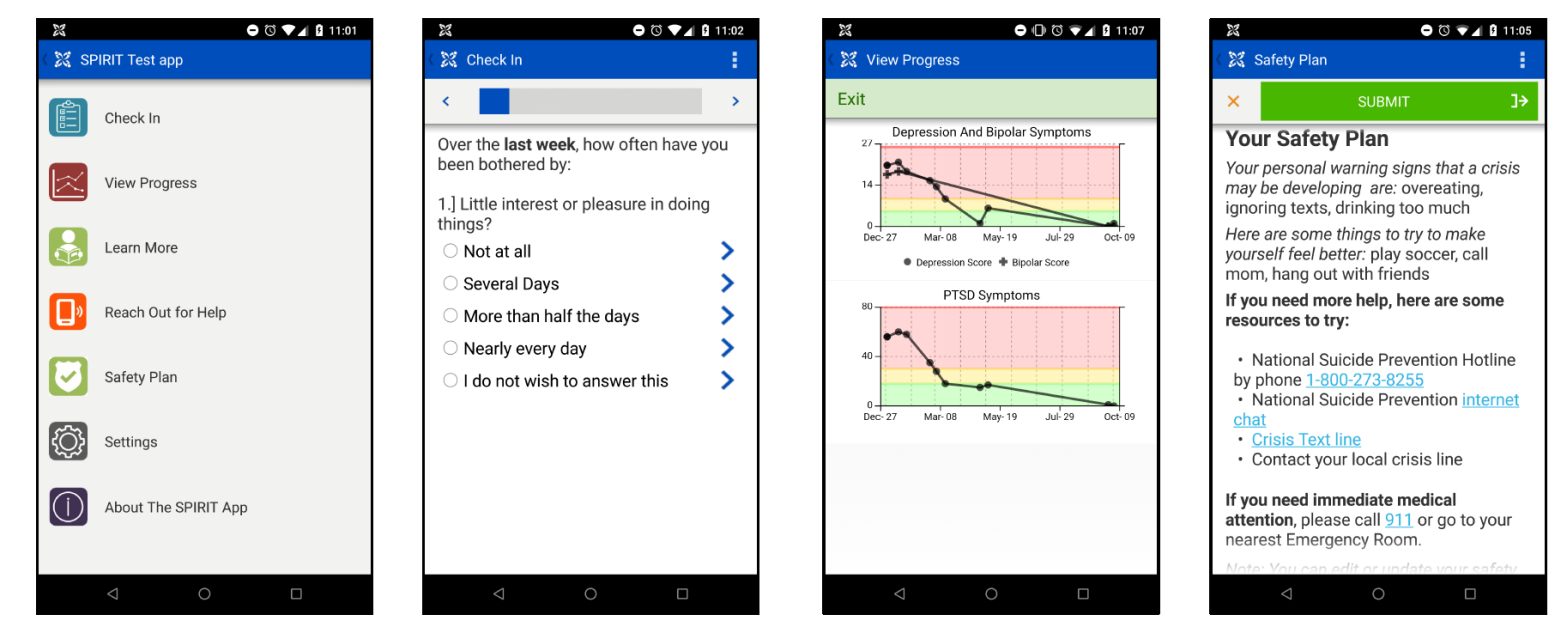
Study to Promote Innovation in Rural Integrated Telepsychiatry (SPIRIT) app screenshots.

The SPIRIT app is organized into seven modules: Check In, View Progress, Learn More, Reach Out for Help, Safety Plan, Settings, and About the SPIRIT App (Figure 3). The Check In module allows patients to self-monitor and report symptoms to their care manager by completing rating scales for depression (PHQ-9), mania (SPIRIT mania scale), PTSD (PTSD Checklist for DSM-5, PCL-5), and medication adherence. In the View Progress module, patients see a graph of their scores to track their own weekly progress over time. The Learn More module has psychoeducational materials about their condition, psychiatric medications, and tips for managing common side effects. It also includes information about how the Collaborative Care model works and Behavioral Activation, a psychotherapy provided by SPIRIT care managers. Patients can access stories from people living with PTSD or bipolar disorder through links to consumer advocacy websites. If patients choose to customize the SPIRIT app, they can use the Reach Out for Help module to make phone calls directly to supportive, self-programmed contacts such as friends, family, or care providers. The Safety Plan module displays general information about how to contact emergency services such as the National Suicide Prevention Lifeline, Crisis Text Line, and Lifeline Chat for all patients regardless of whether or not they enter a personal plan and a personal safety plan for patients who choose to enter one. The Settings module facilitates these customizations and also allows them to personalize their Check In day, the time and message content of short message service (SMS) reminders, and select which symptom scales to report on. They can create personalized reminders to take medications or for recurring activities as part of their Behavioral Activation treatment plan and submit new contact information for their care manager if they change their phone number. The About the SPIRIT App module includes frequently asked questions that explain how the SPIRIT app works and about their data privacy within the system. Patients are prompted to provide feedback through an in-app survey (details provided below).

#### Provider Interface: Care Management Tracking System

The SPIRIT app is directly integrated with CMTS, which care managers use daily in their Collaborative Care workflow to track patient encounters and view progress. To accommodate the SPIRIT app data, we expanded existing CMTS pages to incorporate data from the SPIRIT app and created new features supporting two major functions: (1) registering patients to use the SPIRIT app and manage their account and (2) viewing data their patients enter into the SPIRIT app. To register a patient, a care manager only needs to enter the patient’s mobile phone number into a new CMTS mobile registration page that initiates a patient self-registration process. The patient will then receive an SMS with instructions and a link to download the app. In the CMTS mobile registration page, we embedded links to patient handouts and care manager materials about the SPIRIT app (described below). To view patient data, care managers view an alert on their Reminders page (the first page they see after logging in) to indicate when new app data has been imported for any patient on that care manager’s caseload. If a patient reports suicidal thoughts on the PHQ-9, a specific alert is triggered so that care managers can quickly identify the patient and determine whether additional support such as telephone outreach is indicated. Additional pages in CMTS allow care managers to determine which patients have new scores and view individual item scores for each measure. Thus, SPIRIT app data is integrated throughout CMTS pages and helps care managers visualize app data at the level of the entire caseload, the individual patient, and the encounter.

### App Design Process

The process for designing the SPIRIT app adhered to the Principles for Digital Development and was informed by earlier work with two mobile apps designed for Collaborative Care for depression [19,20]. These principles were created to guide international development organizations, health organizations, and mobile developers in integrating best practices into the design of digital technology-supported service delivery programs. They include nine living guidelines that are intended to be revised over time [20].

**Principles for digital development [19, 20].**

1. Design with the user
2. Understand the existing ecosystem
3. Design for scale
4. Build for sustainability
5. Be data driven
6. Use open standards, open data, open source, and open innovation
7. Reuse and improve
8. Address privacy and security
9. Be collaborative

The current set of principles (Textbox 1) have emerged from a collaborative, community-driven effort that began in the late 2000s with the recognition of struggles with implementation that were faced by digital programs and reflect involvement of diverse organizations such as the World Health Organization, World Bank, United Nations Children’s Fund, the United Nations Development Program, the United States Agency for International Development, the Swedish International Development Agency, and the Bill and Melinda Gates Foundation. The Principles for Digital Development have been endorsed by Dimagi, the organization which developed the SPIRIT app [19].

A detailed description of the steps involved in the SPIRIT app design and development, including proof of concept and other preliminary activities, was previously published [43] and are summarized below (Textbox 2). Service providers such as care managers, health center directors, supervisors, and psychiatrists informed the core content specifications built in CMTS and the SPIRIT app, while patients representative of the final SPIRIT target population were engaged to provide feedback on early concept development, as well as direct feedback and design direction for the app’s user experience, including layout, expected use, new features, and visual design. The project faced challenges with identifying and recruiting a larger cadre of representative patient users to engage in early stage design research, limited by partner health centers’ current active patient list. However, working through the Consumer Advisory Board (CAB) at these early stages was a critical resource in early assessments and research because its members contributed an active interest in communicating patient perspectives in new models to improve mental health care delivery. In the Results section, we illustrate each of the Principles for Digital Development using examples from the SPIRIT app design and development processes.

### Sample and Evaluation Metrics

SPIRIT is a pragmatic clinical trial, and therefore, the procedures and workflow are designed to mirror real-world practice with minimal direct involvement of centralized study staff. Patients receive SPIRIT clinical services for a maximum of 52 weeks. Patients randomized to the Collaborative Care arm of the study can be offered the SPIRIT app by their care manager at any time during the study period. At this time, there are 16 care managers with at least one patient enrolled in the study. We report initial data on the adoption of the SPIRIT app mobile system by five care managers and 10 patients.

Metadata from the CommCare system includes the new user activation date and the time stamp for all data submitted through the SPIRIT app, including each symptom measure. From this mobile metadata, we determined the number of days elapsed since each patient activated the SPIRIT app and the number of symptom measures each patient submitted. Then we calculated the interval of completion of measures as the number of days since activation divided by the number of measures completed. To assess persistence of use, we calculated the number of weeks elapsed between submission of the most recent symptom measure and the date of data extraction.

Patients receive SMS text message notifications to provide feedback through the SPIRIT app at 4, 8, 26, and 40 weeks after activation. The feedback module contains the SUS [46], a widely used 10-item self-report measure of usability for a variety of technologies. Scores range from 0 to 100, and scores above 80 are in the top 10th percentile of usability for all technologies and products tested [47]. Free-text feedback is also elicited in the feedback module. Patients also complete independent research interviews at 6 and 12 months post baseline that include questions about the impact of the SPIRIT app and potential burden. We also ask reasons nonusers did not use the SPIRIT app. Results from these independent research interviews will be reported in a future publication because the SPIRIT study is still ongoing.

**Study to Promote Innovation in Rural Integrated Telepsychiatry (SPIRIT) app design activities.**

Proof of concept phase

- Pilot mixed-methods study of mobile health (mHealth) augmentation of Collaborative Care for depression and anxiety [8]
- Partner with Dimagi to conduct design, development, and usability testing of a depression Collaborative Care app
- Partner with one rural health center (interview one care manager, one patient)
- Integrate depression Collaborative Care app with Care Management Tracking System (CMTS)
- Pilot test linkage between depression Collaborative Care app and CMTS (held focus groups with three care managers; interviews with patient users)

Design and development phase

- Conduct focus group #1 with Study to Promote Innovation in Rural Integrated Telepsychiatry (SPIRIT) Consumer Advisory Board (CAB) to propose initial SPIRIT app concept and gather feedback. CAB comprises a group of individuals who reflect the target user profile and patient population of the SPIRIT deployment
- Conduct focus group #2 with SPIRIT CAB to refine SPIRIT app concept
- Partner with 12 rural community health centers in three states
- Define SPIRIT app design high-level scope
- Build SPIRIT app version 0 high-fidelity prototype and storyboard based on feedback from the CAB and proof of concept user focus groups
- Conduct focus group #3 with SPIRIT CAB to present storyboard of care manager and patient personas, low-fidelity prototype, and elicit feedback
- Refine prototype design; update high fidelity prototype
- Develop insight-driven usability testing framework
- Recruit representative users of the target patient population for usability testing; define participation incentives, consent, and testing logistics
- Conduct usability tests with 5 participants, including patients actively in care at SPIRIT’s health center partner locations [44,45]
- Incorporate feedback from usability testing into app prototype
- Review prototype with programmatic experts, physicians, and study team; elicit and refine feedback
- Finalize SPIRIT app version 1.0

Deployment phase

- Develop patient and provider education materials
- Announce SPIRIT app launch on study website and newsletter and routine calls with study sites
- Launch SPIRIT app in three states
- Host a live webinar demonstration of SPIRIT app for care managers and study investigators
- Foster peer learning using the SPIRIT app in routine care manager training calls
- Invite feedback about how to make improvements in the system or supporting materials, both through patient engagement and an app-supported feedback form
- Provide SPIRIT information and technology (IT) email support for technical issues
- Offer care manager incentives to enroll their first patient
- Develop self-guided training on the SPIRIT app for new care managers when turnover occurs and to onboard new sites

## Results

### Application of Principles for Digital Development

The SPIRIT app design and development illustrates the application of the Principles for Digital Development for technology-enabled service delivery programs and provides new insights on how to optimize these principles within a mental health context [19,20].

#### Principle 1. Design With the End User

The SPIRIT app was created through a human-centered design process that put two groups of end users (patients and providers) at the center of each project stage, including immersion visits to local clinics, user-driven requirements gathering and ideation with multiple stakeholders (such as end users, care managers, supervisors, and consumer advisory groups), 1:1 and group prototype testing and interviews, multiple prototype refinement periods, and implementation driven by local clinics and their care managers [48]. The SPIRIT CAB (consisting of eight consumers and representatives from national consumer advocacy groups), experts in Collaborative Care, and providers and patients in rural clinics were engaged as domain experts [49] and target users in a participatory design process throughout development (Textbox 2). In the postlaunch deployment phase, we continue to obtain feedback from patients through the in-app feedback survey and from clinics and care managers as part of a team-learning process described under *Principle 9: Be Collaborative*. Our longitudinal engagement has yielded improvements in every aspect of the SPIRIT app mobile platform, including the modules of the app, the CMTS provider interface, and our materials and methods to support training and deployment. Further details on specific changes that have been introduced based on stakeholder feedback are provided in the section below, *Incorporation of specific feedback,* and Multimedia Appendix 1.

#### Principle 2. Understand the Existing Ecosystem

Understanding the needs, challenges, and perspectives of Collaborative Care team members, especially the patient, was crucial to developing a mobile tool to extend this care delivery. The research team spent considerable time and effort to understand the context and align the technology with user needs and requirements. Contextual factors included federal law governing health information (ie, HIPAA) and the context of the rural settings, including available infrastructure and mobile phone adoption. Although national data at the time indicated high rates of mobile phone ownership [50], there was a lack of information about device ownership in the specific rural regions that SPIRIT targets. Verifying the nature and prevalence of local mobile phone ownership and connectivity was a key challenge, as was user recruitment for local usability testing given that some of the SPIRIT clinics are small and therefore have few eligible patients. To date, 45.4% (169/372) of patients enrolled in SPIRIT indicated that they own an Android device during the baseline patient survey. Given the uncertainty about device ownership before launch, the SPIRIT CAB encouraged us to consider a multiplatform system that included a patient website and an interactive voice response system in addition to an Android app. We carefully considered a multiplatform system; however, we opted against expanding the scope of the mobile system for this initial demonstration given constraints on time and resources and a desire to prioritize quality by developing a robust system.

#### Principle 3. Define and Plan for Scale

SPIRIT will be the largest clinical trial ever conducted of rural Americans with psychiatric disorders and will provide Collaborative Care services to 500 patients from one of 12 community health centers across Washington, Arkansas, and Michigan. The SPIRIT app links to CMTS, which is used in 22 programs in 24 states throughout the United States and one Canadian province and has helped over 100,000 patients receive better care. The SPIRIT app is available to patients from all of the clinics in the research study. To use the SPIRIT app at large-scale in routine, rural practices, the installation process was simplified and mapped to care managers’ workflows so that patients could install the app independently without direct support from the research team. SPIRIT also developed an SMS-driven self-registration process to explore whether this common communication mechanism is a new approach to helping patients download mHealth apps to their phones privately on their own. This new self-registration process was a key focus for usability testing to ensure that the process was simple. Although the SPIRIT app was designed specifically for use during the study, with minimal modification, the platform could be used at scale in other CMTS implementations, if the experience from SPIRIT supports such expansion.

The SPIRIT app is flexibly designed and is organized into seven modules, as described above. Unlike the depression Collaborative Care app, the SPIRIT app supports multi-condition (depression, bipolar disorder, and/or PTSD) management. The same underlying CommCare platform could be used to support a variety of psychiatric or medical conditions, either by changing the content within the existing modules or by adding or removing modules. As a result, the SPIRIT app is well-positioned for vertical scale to additional clinical sites, as well as horizontal scale into additional psychiatric or medical conditions. Presently, patients select which symptom rating scales to complete; however, it would be possible to enable providers to use CMTS to select rating scales from a menu of choices to tailor care for patients with a variety of conditions. Selecting a mobile platform that enables quick scale-up, reuse, and local adaption was central to SPIRIT development and the team’s decision to select CommCare as the underlying technology platform. CommCare’s evidence base demonstrates the platform’s ability to support both public and private health systems’ to scale mHealth solutions both vertically and horizontally, as well as provide “template” apps that can be used in different programmatic domains.

#### Principle 4. Build for Sustainability

To enable a strong programmatic and technical pathway to sustainability, the SPIRIT app was designed as an augmentation to the Collaborative Care services that are provided within the research study. By integrating with CMTS, the SPIRIT app was built to provide a complementary role within an existing patient management system, in contrast to stand-alone, consumer-facing apps currently dominant in the market. The Patient-Centered Outcomes Research Institute funds SPIRIT and has a strong interest in promoting sustainability of interventions that are supported by research trials. Throughout the SPIRIT study, the research team considered the sustainability of the two clinical care models under study through engagement with policy makers, professional organizations, and health care providers and funders such as the Community Health Plan of Washington, the Michigan Primary Care Association, and Community Health Centers of Arkansas. These partners are actively engaged in developing solutions to challenges such as billing for the clinical services in each of the study arms. To date, sustainability efforts have focused at the higher level of the clinical care models rather than focusing on the specific health technologies to support these. However, as the study progresses, it will be important to include considerations related to the health technologies including CMTS and the SPIRIT app mobile platform. Given that the SPIRIT app is only available for Android devices, it would be important to revisit the possibility of developing a multiplatform system if the platform is implemented for long-term use following the research trial.

#### Principle 5. Be Data Driven

The SPIRIT app development followed a systematic and data-driven design process, especially with regards to usability testing and measuring the effectiveness with which a user could successfully complete key tasks in the app. To understand representative patients’ and care managers’ ease of using the SPIRIT app, our usability testing focused on an insight-driven study of the new self-registration process and users’ ability to navigate to the core components of the SPIRIT app without training. Four essential usability tasks were defined and tested with five representative users. Predefined metrics and goals were established with success thresholds to determine whether or not the functionality tested was usable or not. No more than three pieces of functionality were tested at a single time or contiguously to limit user burden and avoid missing vital feedback at the end of the test. Feedback informed the final SPIRIT app design. Incorporating all high-priority feedback into the app was constrained by a short development time frame and the emergent need to invest more technical resources in expanding the scope of CommCare’s self-registration process, such that we could reduce the number of steps required by a user to successfully register, install, download, and begin using the app. However, this feedback was captured and shared with CommCare’s developers for later in the platform’s roadmap and patient-as-end-user product developments.

A major impetus for the development of the SPIRIT app mobile system was to generate knowledge about how to incorporate a patient-provider mobile platform into the clinical care of patients with complex psychiatric needs in primary care settings. This emphasis on knowledge generation drives a strong commitment to data collection, analysis, and interpretation. Moreover, Collaborative Care is a clinical model based on principles that concurrently emphasize measurement and accountability and patient-centeredness. The evaluation plan for the SPIRIT app mobile system is similarly holistic and includes both qualitative and quantitative elements. Our extensive stakeholder engagement has generated a wealth of insights that have informed this effort, and additional qualitative evaluation from patients will occur via open-ended questions in the in-app feedback survey and through qualitative research interviews that will be conducted with a subset of SPIRIT participants. A variety of quantitative metrics are available through the CommCare platform, and we are now developing reports to aggregate data on SPIRIT app system usage and considering how best to use these results to inform our deployment efforts near real time. Our commitment to knowledge generation has translated into publishing early lessons while the study is still underway [43].

#### Principle 6. Use Open Standards, Open Data, Open Source, and Open Innovation

The SPIRIT app is built on CommCare, an open source mobile data collection platform. The SPIRIT app has both benefitted from and contributed to the platform by providing new requirements necessary to open up the platform to patient-as-end-users and resources to invest in further development. The majority of the features in the SPIRIT app were built upon CommCare’s existing platform functionality such as case management, complex logic and branching algorithms, mobile graphing, and SMS-based reminders and data collection. Several of the unique requirements for SPIRIT necessitated the development of new functions such as the self-registration process via SMS and username self-management features (such as password reset and recovery workflows), and a streamlined download experience and UI that required no training compared with prior CommCare deployments in 50 countries. Both of these were subject to rigorous review during usability testing and, as a result, have contributed new infrastructure to the CommCare platform that is now available open source to other health systems around the world.

#### Principle 7. Reuse and Improve

The SPIRIT app mobile system functions as a patient portal for CMTS, a provider-only registry tool that has been used for nearly a decade to support Collaborative Care. The extensive experience with CMTS as a registry provided a strong foundation for the development of the SPIRIT app system. To leverage and improve upon existing mHealth investments, the SPIRIT app was built on an open source SaaS platform that enables agile software design and rapid prototyping and that has been used and improved through implementations by over 500 organizations in more than 50 countries. We leveraged the strengths of the platform throughout development, as feedback from stakeholders was iteratively integrated into the final design, and the core platform technology supported the breadth of requirements. The SPIRIT app was built from a template design of the depression Collaborative Care app and adapted for new contextual requirements. The flexible, modular design facilitated this adaptation, and a thorough quality assurance process was applied to yield further refinements and improvements. As mentioned previously, the SPIRIT app development also invested resources into the overall platform’s capabilities and underlying architecture, so that future programs and applications can reuse and benefit from these improvements. The development team also conducted a review of common consumer-facing apps available on the app store focused on depression, PTSD, and bipolar self-management. Lessons from these deployments informed the design of the SPIRIT app, such as including Learn More modules to provide easy access to information about specific conditions. To support future design, adaptation, and uptake of mHealth for patient-provider collaboration in mental health care, there is an opportunity for SPIRIT to publish a copy of the SPIRIT app to the CommCare Exchange, an online free marketplace of mHealth template apps that enable other individuals and organizations to download a template app from the Exchange and modify it or use it under the terms of a Creative Commons license.

#### Principle 8. Address Privacy and Security

Protection of patients’ privacy has been a priority throughout the development of the SPIRIT app. Unlike many mobile mental health apps, the SPIRIT app requires a username and password so that patients’ sensitive data is accessible only to authorized users. Data transmission from the SPIRIT app to the CommCare server is encrypted with standards consistent with HIPAA. Both the CommCare platform and CMTS have methods to restrict access to patient identifiers. The self-registration process and patient reminders utilize SMS text messages, and therefore, it is necessary to collect the patients’ mobile phone number, which the care manager also needs to conduct outreach. We minimized the collection of such identifiable information to the minimum necessary and use a CMTS-generated identifier that is separate from a medical record number to link the SPIRIT app data to the CMTS patient record. In response to care manager requests to adapt the SPIRIT app for use on a tablet in the clinic waiting room, we have given careful consideration to the management of patient accounts and workflows to manage the handoff of the device to consecutive patients to prevent accidental access to an incorrect patient account. Patients use the SPIRIT app on an opt-in basis. We have encountered a limitation in communicating with patients about the protections in place for patient privacy and confidentiality. Care managers are the first-line providers who field patient questions; however, they do not have technical expertise in the system and therefore have needed additional support in responding to patients’ technical questions related to data transmission and security.

#### Principle 9. Be Collaborative

Collaboration among multidisciplinary teams is fundamental to the delivery of Collaborative Care, as well as human-centered design [29,51]. The effective use of health information technologies, including mHealth tools, requires an extension of care teams to include experts in domains such as information technologies, software development, and human-centered design [11]. Effective team-based efforts require the development of new relationships and are facilitated by strong communication and empathy. In SPIRIT, we have 30 clinics from 12 organizations with substantial variability in the qualifications and experience of provider teams, the clinical workflows, and the volume and sociodemographic characteristics of patients. The majority of the interactions with sites have been conducted via videoconferencing because sites are in rural areas, and the study investigator team is distributed across several universities in three states. Similarly, many of the activities in developing the SPIRIT app have been conducted remotely and supplemented with site visits and in-person usability testing. We have embraced a team-learning approach to the development and deployment of the SPIRIT app in which care managers partner with us to explore how the SPIRIT app can improve the care they deliver and their patients’ outcomes [52], and patients partner with us to provide direct feedback about what would make a tool more meaningful to use.

### Study to Promote Innovation in Rural Integrated Telepsychiatry App Mobile System Adoption

As of January 25, 2018, five care managers and 10 patients have piloted the SPIRIT app mobile system, and another four patients have been registered by their care manager but have not yet completed the activation process. Patients have had access to the system for an average of 20 weeks (range 1-37 weeks) and submitted an average of 24.1 symptom measures (range 3-52 measures). All 10 patients completed the PHQ-9 and PCL-5 in equal frequencies (mean 9.7, range 1-26 measures). For the seven patients who have completed the SPIRIT mania scale, they have completed it in similar frequencies (mean 6.7, range 1-13 measures). Since activating the SPIRIT app, patients have completed symptom measures on average every 14 days (range 7-63 days). The wide range in the interval of symptom measure completion is accounted for because three patients have not submitted symptom measures for more than 16 weeks. All three of these patients submitted their most recent symptom score within 6 weeks of activating the app, suggesting they stopped using the app around that time point. The remaining seven patients have submitted symptom measures within the 2 weeks preceding data extraction, which is consistent with persistent use of the SPIRIT app. This subset of seven persistent app users have used the system for an average of 16 weeks (range 1-37 weeks) and are submitting symptoms measures on average every 10 days (range 7-16 days).

### Patient Feedback Survey Results

Nine patients activated the app more than 8 weeks before data extraction, which means that they have been prompted at least twice to provide feedback (at week 4 and week 8). Two of the nine patients provided feedback at week 4, both of whom also provided feedback at week 8. Two additional patients provided feedback at week 8 only. Because more patients responded at week 8, we report SUS scores from this time point. Patients rated usability very highly at week 8 (mean 91.9, range 72.5-100).

In the free-text responses, patients reported that they most liked the SPIRIT app’s simplicity, ease of use, convenience, reminders, being in touch with the care team, and prompts to think about oneself. Patients least liked that the symptom measures were a little long and that symptom monitoring was weekly rather than daily. Patients desired to receive feedback from their care team directly through the SPIRIT app. At present, data transmission from CMTS to CommCare is limited to information required for account registration, although two-way communication of clinical information could be developed in the future.

### Incorporation of Specific Feedback

Our extensive, longitudinal engagement with stakeholders resulted in a number of improvements to the SPIRIT app, CMTS provider interface, and deployment processes. For each of these domains, Multimedia Appendix 1 lists specific changes that were introduced based on stakeholder feedback, as well as the project phase and description of the findings that led to the change. Taken together, it is apparent that the iterative processes of obtaining and incorporating feedback occurred throughout project phases and resulted in improvements of every aspect of the system and deployment procedures. Importantly, because the focus of stakeholder engagement evolved over the design and development process, the different project phases provided insights into different aspects of the system such that not all project phases contributed to all of the final system components and procedures.

## Discussion

### Principal Findings

The technology to build mHealth apps to manage mental disorders is increasingly commonplace, yet their integration into clinical care has lagged. The design of the SPIRIT app adhered to the Principles for Digital Development, which address many important issues in the development of technology-enabled services. Early feedback from patients using the SPIRIT app supports its excellent usability in the initial weeks of adoption; however, more feedback is needed from those patients who were offered the SPIRIT app but have not enrolled or have enrolled but have not submitted scores. A majority of SPIRIT app users are self-monitoring their symptoms on average every 2 weeks, which is considerably more frequently than clinic-based symptom assessment. Moreover, it is promising that most of the SPIRIT app users to date (n=7, 70%) have sustained such self-monitoring for 4 or more months.

Despite these encouraging findings, our findings must be interpreted within the context of certain limitations. The SPIRIT app has been developed in the context of a pragmatic research trial designed to mirror real-world rural practice; however, important differences from routine practice remain that could affect the sustainability of the clinical care models. The deployment of novel health technologies into routine clinical practice settings involves additional challenges that must be overcome. These include mapping the technology onto the clinical workflow, while being sensitive to the time investment needed on the part of care providers to fully participate in the design process; educating providers and patients; and developing robust support systems. Moreover, not all providers or patients will embrace digital health tools, even if they are well-designed and integrated into care processes, and further understanding of the limitations of digital mental health tools is needed. In SPIRIT, the overall number of care managers and patients who are currently using the system remains small, as are the response rates to in-app patient feedback surveys. Care managers suggest that lack of Android devices, a barrier we anticipated, has limited SPIRIT app use. This reinforces the notion that a single platform system would be unlikely to be tenable in routine clinical practice. More information is needed about reasons patients discontinue use of the SPIRIT app early in treatment to understand barriers to ongoing use.

Several features have emerged as potential improvements that could be considered in the future. Patients have expressed interest in using the SPIRIT app to communicate directly with their provider. Offering a secure messaging service may be one way to promote sustained use; however, careful consideration would need to be given to educating patients and providers with appropriate expectations for response time and whether the service can be used for crisis management. Patients have also expressed an interest in having a diary function or ability to annotate their symptom scores, and more work is needed to understand how best to structure and aggregate such information and communicate it to care teams. Finally, although the SPIRIT app integrates directly with the Web-based patient registry, neither directly integrates with clinic EHRs. Large-scale adoption of mHealth tools would be facilitated by the development of an integrated suite of health information and technology tools, with patient registry functions to support population health management and patient-facing mobile tools that integrate with EHRs.

As we continue to embark on team learning in collaboration with our clinical partners, we have gathered lessons from these challenges. Our experience with the design, development, and deployment of the SPIRIT app reveals additional considerations for mobile designers and clinical researchers seeking to advance mHealth integration into clinical care. Here we propose five additional principles: design for public health impact, add value for all users, test the product and the process, acknowledge disruption, and anticipate variability.

### Design for Public Health Impact

Although the Principles for Digital Development have been informed by a number of public health efforts, we propose an explicit emphasis on optimizing population health by prioritizing the development of technology-supported services to maximize clinical impact for populations who experience the greatest need. To be truly impactful, digital mental health efforts must not focus exclusively on the most technologically savvy, well-resourced, or least impaired members of society [53]. Numerous studies have found that the benefits realized from efforts to improve the quality of mental health service delivery, such as through Collaborative Care, are greatest among the most disadvantaged patients [54-58]. Yet, just as efforts to improve health care quality have prioritized high-need populations, so too should efforts to implement technology-supported mental health service models that meet the unique needs of these populations. In SPIRIT, both clinical and sociodemographic characteristics contribute to the complexity of the study population. PTSD and bipolar disorder cause more disability than nearly all other health conditions worldwide [59]. Moreover, rural Americans with PTSD and bipolar disorder lack access to effective treatments because of severe shortages and geographic maldistribution of mental health specialists [60,61]. The intersection of high clinical complexity and high unmet need yields high potential for public health impact of mHealth.

### Add Value for All Users

This is an extension of *Principle 1: Design with the User* and is especially relevant for technology-enabled health care services where technology platforms are used by both patients and providers and thus must meet needs for each group of users simultaneously. Most mobile mental health tools are stand-alone tools that aspire to offer value to consumers or patients (primary users) but do not necessarily offer value to clinicians. In contrast, those that are used to augment clinical services, such as the SPIRIT app, also need to provide value to clinicians (secondary users) by performing useful clinical functions. Conversely, extraneous features that lack clear benefit to users should be eliminated or minimized to limit user burden [11,62]. We aligned the SPIRIT app with an effective clinical care model, Collaborative Care, and focused on those functions that support such care [40]. This emphasis was important in defining and narrowing the scope of the app. For example, although efforts to use passive sensing to infer mental health symptoms are emerging, the validity of most measures is not established. Some patients do not want sensor-based data, and it remains unclear how clinicians would use this information to deliver measurement-based care [63]. Therefore, we elected not to include sensor data collection in the scope.

Before deploying the SPIRIT app, we envisioned the SPIRIT app for patients’ use in the community. Subsequently, we received feedback from SPIRIT care managers interested in using the app on a tablet in the waiting room so that patients enter their own symptom scales before appointments. The SPIRIT providers recognized potential value from using the tool in a different workflow than we had anticipated. To provide additional value to care managers, who are key to adoption of the SPIRIT app, we are working on developing necessary workflows and adaptations.

### Test the Product and the Process

We approached quality assurance testing from a technical perspective and focused on specific functions of the SPIRIT app and CMTS linkages. This testing ensured that the registration process, the app, and the data transfer to CMTS were reliable and robust. However, our predeployment testing did not emulate the typical Collaborative Care workflow in which a provider manages a panel of patients and therefore did not serve to pilot test the overall technology-enabled workflow. As such, our testing process was more focused on the technology product than the technology-enabled service process [9]. A more comprehensive testing plan and/or a deployment time line that planned for a soft pilot launch and additional app refinement period would have allowed us to identify improvements in the technology-supported clinical workflow before full-scale launch. For future deployments, we recommend expanding the scope of testing to mirror practice by employing clinicians as testers before deployment during user-acceptance testing and using case-based example patient scenarios, simulation, or role-plays. Such workflow process testing should be accounted for as a separate step in project timelines and budgets for efforts to develop technology-enabled clinical services.

### Acknowledge Disruption

When new technologies are deployed in new clinical settings, some disruption in routines is inevitable, whether or not this is intended. Although individual providers may need to alter their behaviors, implementation is even more challenging when multiple members of the health care team are affected because a new technology requires the development of new clinical workflows [13,14,52]. New technologies can disrupt existing organizational routines and relationships, requiring teams to relearn how to work together [52]. Anticipating, acknowledging, and planning for such disruption can help systems to succeed in efforts to adopt new digital technologies. SPIRIT care managers were already learning new workflows and skills to provide care management and behavioral interventions. We sought to minimize additional burden on care managers as they developed competence working with the SPIRIT app. We did not expect care managers to be experts in the technical details of the system. However, as the front-line clinicians interacting with patients, they have been fielding questions about the SPIRIT app. We developed training materials and resources, including sample scripts, for care managers that were easily accessible to equip them for this new responsibility.

### Anticipate Variability

Some digital health tools may work better in certain clinical settings, and successful adoption of a new digital health technology in one setting may not generalize to other settings. The complex interaction between a new digital technology and the dynamics of the health care team is a key determinant of the success of deployment and may be more important than the features of the technology itself [52]. In SPIRIT, we have 30 clinics from 12 organizations with substantial variability in the qualifications and experience of provider teams, the clinical workflows, the sites’ fidelity to the Collaborative Care clinical model, and the volume and sociodemographic characteristics of patients. All five of the care managers with patients using the SPIRIT app practice in sites that are implementing Collaborative Care well. We anticipate that variability in the interaction between the technology and the dynamics of care teams across sites may lead to greater success with deployment in some clinical contexts than others [52]. This naturally occurring variation provides an additional opportunity for team learning to understand how best to facilitate adoption. Settings also vary in the size and clinical characteristics of the patient populations they serve. Because the practice change required to learn a new system is difficult, this initial hurdle may be overcome more readily if providers believe that the new technology will benefit a majority of their patients and have a “critical mass” of eligible patients. In SPIRIT, this presents a challenge in some small rural clinics serving a handful of patients with PTSD and/or bipolar disorder. We anticipate the need for ongoing efforts to support and promote use of the SPIRIT app in settings with low patient volume or high provider turnover. Integration of mHealth tools into clinical practice may be more successful when a single system can be used to support care for a variety of health conditions or for patients who have multiple chronic illnesses rather than having separate apps for each condition. Tools that support multi-condition management are likewise more patient-centered.

### Conclusions

Adhering to the Principles for Digital Development, we created and deployed an mHealth system to support Collaborative Care for patients with complex psychiatric conditions in rural health centers. Early feedback from patients indicates that the SPIRIT app earns high scores for usability among active users. A majority of the initial patients using the SPIRIT app are self-monitoring symptoms on a biweekly basis over a period of several months, which are intervals that are clinically meaningful for supporting measurement-based care. Our findings come from a pragmatic clinical trial designed to reflect real-world clinical practice, and therefore, these patterns occurred with limited involvement of the research team. Our examples of how the Principles for Digital Development applied to the SPIRIT app can serve as a model for clinical researchers and mobile developers. We propose to extend this framework with five additional principles: design for public health impact, add value for all users, test the product and the process, acknowledge disruption, and anticipate variability. These principles can inform future efforts to improve health care quality and outcomes by integrating mobile tools into clinical care pathways in rural practice settings.

## Appendix

Multimedia Appendix 1 Examples of specific changes to the Study to Promote Innovation in Rural Integrated Telepsychiatry (SPIRIT) mobile platform based on end-user feedback.

## Abbreviations

CAB: Consumer Advisory Board
CMTS: Care Management Tracking System
EHR: electronic health record
HIPAA: Health Insurance Portability and Accountability Act
mHealth: mobile health
PCL-5: PTSD Checklist for DSM-5
PHQ-9: Patient Health Questionnaire-9
PTSD: posttraumatic stress disorder
SaaS: Software-as-a-Service
SMS: short message service
SPIRIT: Study to Promote Innovation in Rural Telepsychiatry
SUS: System Usability Scale
UI: user interface
