# Supplementary material for: Applying the Principles for Digital Development: Case Study of a Smartphone App to Support Collaborative Care for Rural Patients With Posttraumatic Stress Disorder or Bipolar Disorder
Source: J Med Internet Res. 2018 Jun 6;20(6):e10048. doi: 10.2196/10048 (PMC6010837; doi:10.2196/10048)
Supplement: Multimedia Appendix 1 [file jmir_v20i6e10048_app1.pdf]

Multimedia Appendix. Examples of specific changes to the Study to Promote Innovation in Rural Integrated Telepsychiatry (SPIRIT) mobile platform based on end-user feedback.

Domain	Project Phase	Findings	Changes
**Overall SPIRIT App Concept**	Development and usability testing	Cellular coverage can be sparse in rural areas. Some clinics have limited network access. The installation time period ranged between 5-25 min depending on network. Download and installation time was unacceptably long in some settings.	To minimize download and installation time, we reduced the volume of multimedia content in the SPIRIT App.
**SPIRIT App Check In module**	Proof-of-concept pilot study of mHealth augmentation of Collaborative Care [1]	Patients desired help interpreting what their symptom rating scale scores mean.	We developed and improved messaging about symptom score interpretation to aid patient self-monitoring.
	Proof-of-concept pilot study of mHealth augmentation of Collaborative Care [1]; proof of concept pilot test of linkage between depression Collaborative Care app and CMTS; and usability testing	Patients and care managers affirmed that patient safety is a top priority and suggested enhancing the App’s messaging to patients in crisis.	We improved in-app messaging when suicidal thoughts are reported to inform patients that scores submitted are not viewed in real-time and direct patients in crisis to use national hotlines or contact their social support system or providers directly by phone.

**SPIRIT App View Progress module**	Proof-of-concept pilot study of mHealth augmentation of Collaborative Care [1]	Patients requested a feedback graph of their scores.	We created graphs to track symptom scores and encourage patients to self-monitor symptoms.
	Usability testing	Some patients expressed concern that the red color used to display high symptom scores may be disturbing to patients who already knew they were doing poorly.	To represent high scores sensitively and avoid demoralizing patients, we refined the graphs and muted the colors scheme (Figure 3).
**SPIRIT App Learn More module**	Development and usability testing	Consumer Advisory Board members and patients desired to learn more from other people with the same conditions.	We added a broader range of educational materials and links to stories from people with lived experience of PTSD and/or bipolar disorder.
**SPIRIT App Safety Plan module**	Usability testing	Care managers desired to have the SPIRIT App provide individualized support for patients in crisis.	We created a new suicide safety plan module that mirrors safety plans used in clinical practice (Figure 3).
**SPIRIT App Settings module**	Proof-of-concept pilot study of mHealth augmentation of Collaborative Care [1]	Patients requested more customization, including when they receive notifications, and what the notifications say.	We created several optional customizations to give patients a more personalized experience. These include selecting the time and day for symptom check-ins, selecting which

			symptoms to monitor, and entering customized language for prompts to protect privacy. Patients can also develop a personal safety plan.
**About the SPIRIT App module**	Post-launch deployment phase	Care managers submitted questions to the research team that patients asked about the type of data the SPIRIT App collects.	We updated FAQs based on questions from patients and care managers about data collection and security.
	Post-launch deployment phase	In-App feedback surveys have been completed by four of the nine patients who have been prompted.	To promote completion of the feedback survey, we added follow-up reminders to patients who do not complete the survey after the initial prompt. We also improved the messaging about how to navigate to the feedback survey module to make it easier for patients to locate it.
**CMTS Provider view**	Proof-of-concept pilot study of mHealth augmentation of Collaborative Care [1]	The care manager indicated that accessing a separate provider dashboard was a limitation.	The SPIRIT App links directly to CMTS.
	Proof of concept pilot test of linkage between depression	Locating SPIRIT App data in CMTS was difficult for care managers.	We integrated SPIRIT App data into multiple CMTS pages to visualize data at

	Collaborative Care app and CMTS		the level of the entire caseload, individual patient, and encounter.
	Proof of concept pilot test of linkage between depression Collaborative Care app and CMTS	Care managers requested alerts for new App data, especially suicidal thoughts.	We created alerts for App data and for suicidal thoughts that are visible on the first page care managers see upon login.
	Deployment phase	The alert for suicide thoughts on the PHQ-9 does not link directly to the record that triggered the alert.	To make it easier for care managers to identify the triggering patient and encounter, we updated CMTS to display scores on the suicide thoughts item.
**Deployment strategies**	Proof-of-concept pilot study of mHealth augmentation of Collaborative Care [1]	Patients had variable understanding of the role of the App in their care.	We created patient education materials that are accessible within CMTS and the SPIRIT project website and brief scripts for care managers to talk to their patients about using the SPIRIT App. We added introductory language in the Patient Set-up form.
	Deployment phase	Care managers in SPIRIT varied in how quickly they learned new clinical skills to provide Collaborative Care. They vary widely in their prior experience and	Because the SPIRIT App is an optional tool, we promoted and encouraged its use gradually, as care managers mastered clinical competencies. We offer

		comfort with behavioral health and with technology. Even for care managers with significant experience with complex psychiatric conditions, using mHealth to support care is new. Some care managers were not comfortable asking their patients to use the SPIRIT App until they had tried it themselves.	test patient accounts to care managers to try the SPIRIT App on their own so they can gain confidence and familiarity. We are providing clinics with a tablet and a test account so care managers without Android phones can use the SPIRIT App and show it to patients.
	Deployment phase	Care managers expressed interest in offering the SPIRIT App to patients in the waiting room prior to visits.	We are working with care managers to develop procedures for managing accounts and workflow for patients to use the SPIRIT App on a tablet. We enhanced CMTS so that care managers can import rating scale scores in real-time.

[1] Bauer AM, Iles-Shih M, Ghomi RH, Rue T, Grover T, Kincler N, Miller M, Katon WJ. Acceptability of mHealth augmentation of Collaborative Care: A mixed methods pilot study. Gen Hosp Psychiatry. 2018;51:22-9. PMID: 29272712. [doi: 10.1016/j.genhosppsych.2017.11.010] [Medline: 29272712]
